# Influence of the selective antagonist of the NR2B subunit of the NMDA receptor, traxoprodil, on the antidepressant-like activity of desipramine, paroxetine, milnacipran, and bupropion in mice

**DOI:** 10.1007/s00702-016-1657-8

**Published:** 2016-11-29

**Authors:** Weronika Stasiuk, Aleksandra Szopa, Anna Serefko, Elżbieta Wyska, Katarzyna Świąder, Jarosław Dudka, Piotr Wlaź, Ewa Poleszak

**Affiliations:** 1 Department of Human Physiology, Medical University of Lublin, Radziwiłłowska 11, PL-20080 Lublin, Poland; 2 Department of Applied Pharmacy, Medical University of Lublin, Chodźki 1, PL-20093 Lublin, Poland; 3 Department of Pharmacokinetics and Physical Pharmacy, Collegium Medicum, Jagiellonian University, Medyczna 9, PL-30688 Kraków, Poland; 4 Department of Toxicology, Medical University of Lublin, Chodźki 8, PL-20093 Lublin, Poland; 5 Independent Medical Biology Unit, Medical University of Lublin, Jaczewskiego 8, PL-20950 Lublin, Poland; 6 Department of Animal Physiology, Institute of Biology and Biochemistry, Faculty of Biology and Biotechnology, Maria Curie-Skłodowska University, Akademicka 19, PL-20033 Lublin, Poland

**Keywords:** Traxoprodil, Antidepressants, Forced swim test, Pharmacokinetic study, NMDA receptor ligand, Mice

## Abstract

Pre-clinical and clinical studies indicated that a blockade of the NMDA receptor complex creates new opportunities for the treatment of affective disorders, including depression. The aim of the present study was to assess the influence of traxoprodil (10 mg/kg) on the activity of desipramine (10 mg/kg), paroxetine (0.5 mg/kg), milnacipran (1.25 mg/kg), and bupropion (10 mg/kg), each at sub-therapeutic doses. Moreover, brain levels of traxoprodil and tested agents were determined using HPLC. The obtained results were used to ascertain the nature of occurring interaction between traxoprodil and studied antidepressants. The experiment was carried out on naïve adult male Albino Swiss mice. Traxoprodil and other tested drugs were administered intraperitoneally. The influence of traxoprodil on the activity of selected antidepressants was evaluated in forced swim test (FST). Locomotor activity was estimated to exclude false positive/negative data. To assess the influence of traxoprodil on the concentration of used antidepressants, their levels were determined in murine brains using HPLC. Results indicated that traxoprodil potentiated activity of all antidepressants examined in FST and the observed effects were not due to the increase in locomotor activity. Only in the case of co-administration of traxoprodil and bupropion, increased bupropion concentrations in brain tissue were observed. All tested agents increased the traxoprodil levels in the brain. Administration of a sub-active dose of traxoprodil with antidepressants from different chemical groups, which act via enhancing monoaminergic transduction, caused the antidepressant-like effect in FST in mice. The interactions of traxoprodil with desipramine, paroxetine, milnacipran, and bupropion occur, at least partially, in the pharmacokinetic phase.

## Introduction

One of the new approaches to the treatment of depression is focused on glutamatergic neurotransmission. It has been shown that a blockade of the NMDA receptor complex creates new opportunities for the treatment of affective disorders (Diazgranados et al. 2010; Gogas 2006; Hashimoto 2011; Permoda-Osip and Rybakowski 2011; Szewczyk et al. 2012). Previous preclinical studies indicate that the NMDA receptor ligands (e.g., CGP 37849, CGP 39551, MK-801, kynurenic acid, 7-chlorokynurenic acid, zinc, magnesium) possess antidepressant-like activity in animal tests and models of depression (Eby and Eby 2010; Maj et al. 1992a, b; Nowak et al. 2003; Poleszak et al. 2005, 2011; Szewczyk et al. 2009), whereas ketamine and memantine are already used successfully in humans (Berman et al. 2000; Keck et al. 2009; Zarate et al. 2006). However, serious side effects such as memory loss, ataxia, and an increase in motor activity related to the application of certain ligands of the NMDA receptor precludes its therapeutic use in humans (Willetts et al. 1990).

Traxoprodil (CP-101,606), an antagonist of NR2B subunit of the NMDA receptor, causes fewer adverse effects in patients, because it does not influence on the α_1_-adrenergic receptors (Chazot et al. 2002; Menniti et al. 1997; Mony et al. 2009). In a study conducted by Guscott et al. the antidepressant activity of two NMDA receptor ligands, MK-801 and traxoprodil, were compared. The results of this study have indicated that traxoprodil has a greater therapeutic potential with no adverse effects on the learning and memory (Guscott et al. 2003). Literature data have shown promising results that confirm the usefulness of traxoprodil in the treatment of depression, and in one study the rapid improvement of mental health in patients who were previously treated unsuccessfully with paroxetine has been described (Preskorn et al. 2008).

It has been shown that NMDA receptor ligands potentiate the effects of conventionally used antidepressant medications (Cieślik et al. 2007; Poleszak et al. 2011; 2013, 2016; Siwek et al. 2009; Stasiuk et al. 2016; Wlaź et al. 2011). The present study is a follow-up to our previous research in which the effect of traxoprodil on the activity of commonly used antidepressants (Poleszak et al. 2016), and drugs with atypical mechanism of action, i.e., mianserin, tianeptine and agomelatine (Stasiuk et al. 2016) were assessed. Therefore, the main goal of this study was to assess the effect of traxoprodil, at the inactive dose, on the activity of antidepressant drugs acting through diverse mechanisms, i.e., desipramine—the tricyclic antidepressant (TCA), paroxetine—a selective serotonin reuptake inhibitor (SSRI), milnacipran selective serotonin and norepinephrine reuptake inhibitor (SNRI), and bupropion—a norepinephrine–dopamine reuptake inhibitor (NDRI) in forced swim test (FST) in mice. Additionally, to evaluate whether the observed animals’ behavior effects were consequent to a pharmacokinetic or pharmacodynamic interaction, concentrations of the studied drugs in murine brain tissue were measured using high-performance liquid chromatography (HPLC).

## Materials and methods

### Animals

The experiments were carried out on naïve adult male Albino Swiss mice (25–30 g) purchased from the licensed breeder (Kołacz, Warsaw, Poland). The total number of animals used in the study was 252. The animals were housed in the environmentally controlled rooms with a 12 h light/dark cycle, in groups of 10 in standard cages under strictly controlled laboratory conditions: temperature maintained at 22–23 °C and relative humidity of 45–55%. Throughout the study, the animals were given ad libitum access to water and food. The experiments began after at least 1-week acclimation period in the laboratory conditions and were conducted between 8 am and 3 pm to minimize circadian influence. Each experimental group consisted of 8–10 animals. All procedures were conducted in accordance with the European Communities Council Directive of 22 September 2010 (2010/63/EU) and Polish legislation acts concerning animal experimentations. The experimental procedures and protocols were approved by the First Local Ethics Committee at the Medical University of Lublin (license no 33/2013). Each mouse was used only once.

### Drug administration

Traxoprodil (10 mg/kg, Sigma-Aldrich) was suspended in a 1% aqueous solution of Tween 80 (POCH), whereas desipramine hydrochloride (10 mg/kg, Sigma-Aldrich), paroxetine hydrochloride (0.5 mg/kg, Sigma-Aldrich), milnacipran hydrochloride (1.25 mg/kg, Abcam Biochemicals), and bupropion hydrochloride (10 mg/kg, Abcam Biochemicals), were dissolved in physiological saline (0.9% NaCl). The solutions/suspension were prepared immediately prior to the experiments and were administered intraperitoneally (i.p.) 60 min before testing. The doses and pretreatment schedules were selected on the basis of the literature data and the results of our previous experiments (Peng et al. 2007; Piotrowska et al. 2013; Poleszak et al. 2015; Szopa et al. 2016). Animals from the control groups received i.p. injections of the vehicle (saline). The volume of all administered solutions/suspension was 10 ml/kg.

### Forced swim test (FST)

The procedure was carried out on mice, according to the method of Porsolt et al. (1977). Each mouse was placed individually into a glass cylinder (height 25 cm, diameter 10 cm) containing 12–15 cm of water at 23–25 °C. The animal was left in the cylinder for 6 min. The total duration of immobility was recorded during the last 4 min of the 6-min testing period. The mouse was judged to be immobile when it ceased struggling and remained floating motionless in the water, making only the movements necessary to keep its head above the water level.

The results obtained in the FST were shown as an arithmetic mean of immobility time of animals (given in seconds) ± standard error of the mean (SEM) for each experimental group.

### Spontaneous locomotor activity

To avoid the risk of obtaining the false positive/negative effects in the FST caused by a possible influence of the tested drugs on the locomotor activity, spontaneous locomotor activity was measured using an animal activity meter Opto-Varimex-4 Auto-Track (Columbus Instruments, USA). The device consists of four transparent cages with a lid (43 × 43 × 32 cm), a set of four infrared emitters (each emitter has 16 laser beams), and four detectors monitoring animal movements. Each mouse was placed individually into the cage for 10 min. Spontaneous locomotor activity was evaluated between the 2nd and the 6th min, which corresponds with the time interval analyzed in the FST.

The results obtained in this test were presented as an arithmetic average distance (given in cm) traveled by a mouse ± SEM for each experimental group.

### Determination of antidepressants’ levels in brain tissue

Sixty minutes following administration of studied antidepressant drugs with or without traxoprodil, mice were decapitated to collect brains for pharmacokinetic studies. Immediately after the decapitation, the brains were dissected from the skull, washed with 0.9% NaCl and frozen at −25 °C.

Brain concentrations of the studied antidepressants were assayed by a HPLC method. The brains were homogenized in distilled water (1:4, w/v) with a tissue homogenizer TH220 (Omni International, Inc., Warrenton, VA, USA). For all studied drugs, the extraction from brain homogenates were performed using the mixture of ethyl acetate:hexane (30:70, v/v). The exceptions were paroxetine, for which the solvents were mixed at a 50:50, v/v ratio. Amitriptyline (2 μg/ml) was used as an internal standard (IS) for desipramine, desipramine (500 ng/ml) for paroxetine, bupropion (1 μg/ml) for milnacipran, and milnacipran (1 μg/ml) for bupropion.

In order to isolate desipramine, to brain homogenate (0.5 ml) containing this drug IS was added and the samples were alkalized with 250 μl of 4 M NaOH. Next, the samples were extracted with 5 ml of the extraction reagent by shaking for 20 min (IKA Vibrax VXR, Germany). After centrifugation at 3000 rpm for 20 min (Universal 32, Hettich, Germany), the organic layer was transferred to a new tube containing a 200 μl solution of 0.1 M H_2_SO_4_ and methanol (90:10, v/v), shaken for 0.5 h and then centrifuged for 15 min (3000 rpm). Then the organic layer was discarded and a 50 μl aliquot of acidic solution was injected into the HPLC system. A similar extraction procedure was applied for milnacipran and bupropion with the exception that 1 ml of brain homogenate was used and the drugs were reextracted to 100 μl of 0.1 M HCl.

Paroxetine was extracted from 1 ml of brain homogenate. After the addition of IS and 250 μl of 4 M NaOH, 1 ml of the concentrated NaCl solution (10 g/50 ml) was added and the samples were vortexed for 15 s. Then 5 ml of the extraction reagent was added and the samples were shaken for 20 min and centrifuged for 15 min at 3000 rpm. After the centrifugation, the organic layer was transferred into a conical glass tube and evaporated to dryness at 37 °C under a gentle stream of nitrogen in a water bath. The residue was dissolved with 100 μl of methanol and 50 μl of this solution was injected into the HPLC system.

The HPLC system (Thermo Separation Products, San Jose, CA, USA) consisted of a P100 isocratic pump, a UV100 variable-wavelength UV/VIS detector, a Rheodyne 7125 injector (Rheodyne, Cotati, CA, USA) with a 50 μl sample loop, and a Chromjet SP4400 computing integrator.

All analyses were performed on a 250 × 4.0 mm LiChrospher^®^100 RP-18 column with a particle size of 5 μm (Merck, Darmstadt, Germany) protected with a guard column (4 × 4 mm) with the same packing material. The mobile phase consisting of acetonitrile and 50 mM potassium dihydrogen phosphate was mixed at a ratio of 40:60 (v/v) for desipramine, 35:65 (v/v) for paroxetine, 25:75 (v/v) for milnacipran and bupropion, and run at 1 ml/min. Chromatographic analysis was carried out at 21 °C and the analytical wavelength of 200 nm for milnacipran and 214 nm for the other studied antidepressant drugs.

To determine traxoprodil concentrations in mice brain, to 1 ml of brain homogenate containing this compound 2 ml of methanol was added and the samples were briefly vortexed and then shaken vigorously for 10 min (IKA Vibrax VXR, Germany) to precipitate proteins. After centrifugation for 20 min at 3000 rpm the supernatant (2 ml) was transferred into a conical glass tube and evaporated to dryness at 45 °C under a gentle stream of nitrogen in a water bath. The residue was dissolved with 100 μl of methanol and 40 μl of this solution were injected into the HPLC system.

The HPLC system (Merck-Hitachi LaChrom Elite) consisted of an L-2130 pump, an L-2200 autosampler, an L-2350 column oven, and an L-2485 fluorescence detector. EZChrome Elite v. 3.2 (Merck Hitachi) software was used for data acquisition. The analysis was performed on a 250 × 4.0 mm LiChrospher^®^100 RP-18 column (Merck, Darmstadt, Germany) maintained at 30 °C, protected with a guard column (4 × 4 mm) of the same material. The mobile phase consisted of 50 mM potassium dihydrogen phosphate buffer (pH 4.5):acetonitrile:methanol (70:20:10, v/v/v). The flow rate was 1.0 ml/min and the fluorescence detector was set at an excitation wavelength of 200 nm and an emission wavelength of 300 nm.

The calibration curves constructed by plotting the ratio of the peak heights of the studied drug to IS (or peak area for traxoprodil) versus concentration of the drug were linear in the tested concentration ranges. No interfering peaks were observed in the chromatograms. The assays were reproducible with low intra- and inter-day variation (coefficient of variation less than 10%). The extraction efficiencies of the analyzed compounds and internal standards ranged from 66 to 97%. Concentrations of antidepressants and traxoprodil were expressed in ng/g of wet brain tissue.

### Statistical analysis

The statistical analysis of the results obtained in the FST and locomotor activity was done using two-way ANOVA with Bonferroni’s post hoc test. The concentrations of the tested antidepressant drugs in murine brains in the presence and absence of traxoprodil were compared using Student’s *t* test. *P* values less than or equal to 0.05 were considered statistically significant.

## Results

### Forced swim test (FST)

#### Effect of combined administration of traxoprodil and desipramine in FST

**Fig. 1:**
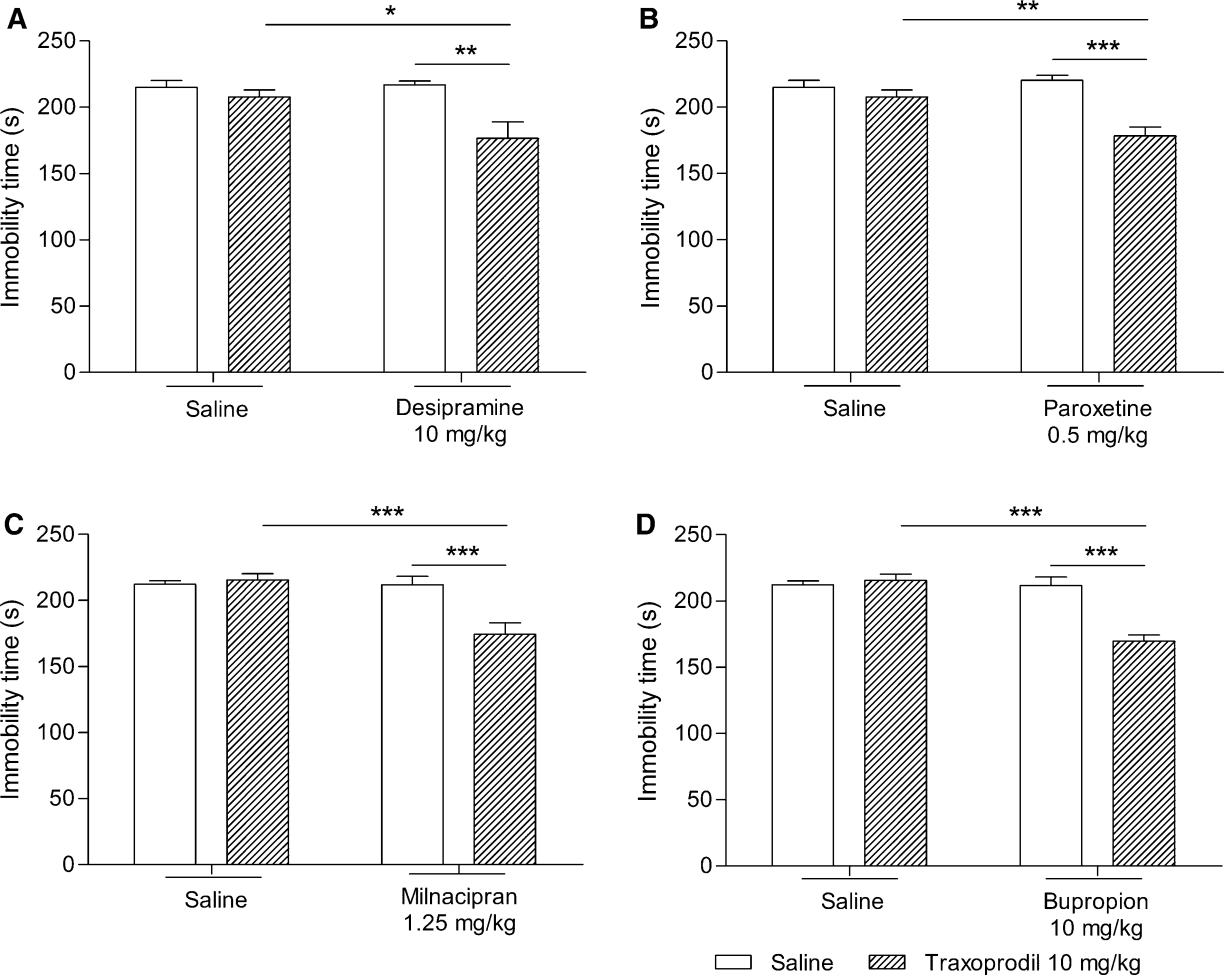
Effect of combined administration of traxoprodil and antidepressants in the FST in mice. Antidepressants, traxoprodil and saline were administered i.p. 60 min before the test. The values represent mean ± SEM (*n* = 10 per group). **p < 0.01; ***p < 0.001 (two-way ANOVA followed by Bonferroni’s post hoc test)

The effect of the combined administration of traxoprodil and desipramine on total duration of the immobility time in mice is shown in Fig. 1a. Traxoprodil (10 mg/kg) injected in combination with desipramine (10 mg/kg) significantly reduced the immobility time in the FST in mice (Fig. 1a). Desipramine (10 mg/kg) and traxoprodil (10 mg/kg) given alone had no effect on the immobility time (Fig. 1a).

Two-way ANOVA demonstrated a significant effect of traxoprodil [*F*(1,28) = 10.32; *p* = 0.0033], no effect of desipramine [*F*(1,28) = 3.93; *p* = 0.0574], and a significant interaction between desipramine and traxoprodil [*F*(1,28) = 4.99; *p* = 0.0336].

#### Effect of combined administration of traxoprodil and paroxetine in FST

The effect of the combined administration of traxoprodil and paroxetine on total duration of the immobility time in mice is shown in Fig. 1b. Traxoprodil (10 mg/kg) injected in combination with paroxetine (0.5 mg/kg) significantly reduced the immobility time in the FST in mice (Fig. 1b). Paroxetine (0.5 mg/kg) and traxoprodil (10 mg/kg) given alone had no effect on the immobility time (Fig. 1b).

Two-way ANOVA demonstrated a significant effect of traxoprodil [*F*(1,28) = 21.15; *p* < 0.0001], a significant effect of paroxetine [*F*(1,28) = 5.07; *p* = 0.0323], and a significant interaction between paroxetine and traxoprodil [*F*(1,28) = 10.49; *p* = 0.0031].

#### Effect of combined administration of traxoprodil and milnacipran in FST

The effect of the combined administration of traxoprodil and milnacipran on total duration of the immobility time in mice is shown in Fig. 1c. Traxoprodil (10 mg/kg) injected in combination with milnacipran (1.25 mg/kg) significantly reduced the immobility time in the FST in mice (Fig. 1c). Milnacipran (1.25 mg/kg) and traxoprodil (10 mg/kg) given alone had no effect on the immobility time (Fig. 1c).

Two-way ANOVA demonstrated a significant effect of traxoprodil [*F*(1,36) = 7.66; *p* = 0.0089], a significant effect of milnacipran [*F*(1,36) = 11.36; *p* < 0.0018], and a significant interaction between milnacipran and traxoprodil [*F*(1,36) = 11.14; *p* = 0.0020].

#### Effect of combined administration of traxoprodil and bupropion in FST

The effect of the combined administration of traxoprodil and bupropion on total duration of the immobility time in mice is shown in Fig. 1d. Traxoprodil (10 mg/kg) injected in combination with bupropion (10 mg/kg) significantly reduced the immobility time in the FST in mice (Fig. 1d). Bupropion (10 mg/kg) and traxoprodil (10 mg/kg) given alone had no effect on the immobility time (Fig. 1d).

Two-way ANOVA demonstrated a significant effect of traxoprodil [*F*(1,36) = 16.21; *p* = 0.0003], a significant effect of bupropion [*F*(1,36) = 23.21; *p* < 0.0001], and a significant interaction between bupropion and traxoprodil [*F*(1,36) = 22.61; *p* < 0.0001].

### Spontaneous locomotor activity

#### Effect of combined administration of traxoprodil and antidepressants on locomotor activity in mice

The effect of the combined administration of traxoprodil and tested antidepressant drugs on spontaneous locomotor activity in mice is shown in Table 1.

**Table 1:** Effect of treatments on spontaneous locomotor activity in mice

	Treatment (mg/kg)	Distance traveled (cm)
(A)	Saline + saline	652.9 ± 54.25
	Traxoprodil 10 + saline	586.4 ± 63.26
	Desipramine 10 + saline	575.8 ± 77.27
	Traxoprodil 10 + desipramine 10	518.3 ± 35.29
(B)	Saline + saline	766.9 ± 41.48
	Traxoprodil 10 + saline	755.6 ± 59.53
	Paroxetine 0.5 + saline	745.8 ± 75.61
	Traxoprodil 10 + paroxetine 0.5	796.9 ± 88.63
(C)	Saline + saline	680.0 ± 83.19
	Traxoprodil 10 + saline	679.8 ± 49.99
	Milnacipran 1.25 + saline	652.9 ± 74.75
	Traxoprodil 10 + milnacipran 1.25	757.0 ± 94.51
(D)	Saline + saline	680.0 ± 83.19
	Traxoprodil 10 + saline	679.8 ± 49.99
	Bupropion 10 + saline	742.3 ± 79.57
	Traxoprodil 10 + bupropion 10	969.3 ± 64.82

Antidepressants, traxoprodil and saline were administered i.p. 60 min before the experiment. Distance traveled was recorded between the 2nd and the 6th min of the test. Each experimental group consisted of 8 animals. Data are presented as the mean ± SEM. The results were considered statistically significant if *p* < 0.05 (two-way ANOVA followed by Bonferroni’s post hoc test)

Traxoprodil (10 mg/kg) and all tested antidepressants (desipramine, paroxetine, milnacipran, bupropion) administered either alone or combined together had no statistically significant effects on locomotor activity in mice (Table 1).

Two-way ANOVA demonstrated:

1. No effect of desipramine [*F*(1,27) = 2.49; *p* = 0.1265], no effect of traxoprodil [*F*(1,27) = 1.98; *p* = 0.1709], and no interaction [*F*(1,27) = 0.24; *p* = 0.6304].
2. No effect of paroxetine [*F*(1,28) = 0.02; *p* = 0.8845], no effect of traxoprodil [*F*(1,28) = 0.08; *p* = 0.7735], and no interaction [*F*(1,28) = 0.21; *p* = 0.6530].
3. No effect of milnacipran [*F*(1,27) = 0.00; *p* = 0.9489], no effect of traxoprodil [*F*(1,27) = 0.86; *p* = 0.3608], and no interaction [*F*(1,27) = 0.17; *p* = 0.6814].
4. No effect of bupropion [*F*(1,26) = 3.73; *p* = 0.0644], no effect of traxoprodil [*F*(1,26) = 2.55; *p* = 0.1225], and no interaction [*F*(1,26) = 1.00; *p* = 0.3266].

### Pharmacokinetic studies

The effect of traxoprodil on brain concentrations of the tested antidepressants in mice brain is shown in Table 2. A significant increase in the concentrations of bupropion in brain tissue after joint administration with traxoprodil was noticed (*t* test: *p* < 0.01). In the case of co-administration of traxoprodil and the other tested drugs, no significant changes in their concentrations in brain were observed (*t* test: *p* > 0.05) (Table 2).

**Table 2:** Effect of traxoprodil on the concentration of antidepressants in mouse brain

Treatment (mg/kg)		Antidepressants concentration in brain (ng/g)
(A)	Desipramine 10 + saline	1649 ± 96.15
	Desipramine 10 + traxoprodil 10	2217 ± 293.1
(B)	Paroxetine 0.5 + saline	140.0 ± 8.823
	Paroxetine 0.5 + traxoprodil 10	150.5 ± 8.733
(C)	Milnacipran 1.25 + saline	410.6 ± 80.60
	Milnacipran 1.25 + traxoprodil 10	337.7 ± 38.28
(D)	Bupropion 10 + saline	965.7 ± 74.02
	Bupropion 10 + traxoprodil 10	1566 ± 249.6**

Antidepressants and traxoprodil were administered i.p. 60 min before decapitation. Each experimental group consisted of 10 animals. Results are presented as mean values ± SEM. The results were considered statistically significant if *p* < 0.05

** *p* < 0.01 compared with the respective control group (Student’s *t* test)

The effect of tested drugs on brain concentrations of traxoprodil in mice is shown in Table 3. In the case of joint administration of traxoprodil and desipramine, paroxetine, milnacipran or bupropion a significant increase in traxoprodil concentrations in brain was noted (*p* = 0.0006, *p* = 0.0003, *p* < 0.0001, *p* < 0.0001, and *p* = 0.0012, respectively, *t* test).

**Table 3:** Effect of antidepressants on the concentration of traxoprodil in mouse brain

Treatment (mg/kg)	Traxoprodil concentration in brain (ng/g)
Traxoprodil 10 + saline	76.40 ± 13.51
Traxoprodil 10 + desipramine 10	253.0 ± 37.92***
Traxoprodil 10 + paroxetine 0.5	271.7 ± 39.33***
Traxoprodil 10 + milnacipran 1.25	500.7 ± 73.14***
Traxoprodil 10 + bupropion 10	303.2 ± 33.88***

Antidepressants and traxoprodil were administered i.p. 60 min before decapitation. Each experimental group consisted of 8 animals. Results are presented as mean values ± SEM. The results were considered statistically significant if *p* < 0.05

*** *p* < 0.001 compared with the control group (Student’s *t* test)

## Discussion

In recent years, a lot of data concerning the influence of glutamate on the effects of antidepressants and mood stabilizers have been gathered. These data indicate that anti-depressants inhibit glutamate system by decreasing the release of glutamate by neurons both in the prefrontal cortex (Michael-Titus et al. 2000) and in the hippocampus (Bonanno et al. 2005; Pittaluga et al. 2007). Perhaps one of the most important mechanisms of action of antidepressants is the glutamate effect on NMDA receptors, which manifests in a reduced expression and function of these receptors leading to the occurrence of its adaptive changes (Nowak et al. 1995). Some research demonstrated that administration of tricyclic antidepressants (TCAs, imipramine), serotonin reuptake inhibitors (SSRIs, fluoxetine), selective noradrenaline reuptake inhibitors (SNRIs, reboxetine), and monoamine oxidase inhibitors (MAOI) leads to the impairment of the function of NMDA receptors (Pittaluga et al. 2007; Skolnick et al. 1996). Antidepressant drugs acting through serotonergic or glutamatergic neurotransmission seem to manifest different biological properties. For example, the indole-3-pyruvic acid, which is metabolized to kynurenic acid was capable to normalize the endocrine dysregulation observed during the depression, while reversing of the behavioral responses associated with depression was not observed (Biagini et al. 1993). In this regard, the indole-3-pyruvic acid was superior to imipramine in the protection of the adrenal hyperactivation in animals. Therefore, it seems that drugs acting on serotonin transduction may compensate the poor ability of glutamate antagonists to regulate behavioral responses. Moreover, the antagonism of the glutamate receptor may enhance the capability of serotonergic drugs to prevent the consequences of chronic stress.

In the present study, the NR2B subunit selective NMDA antagonist, traxoprodil, co-administered with agents which affect monoaminergic neurotransmission at inactive doses, produced a significant antidepressant-like effect in the forced swim test in mice. The synergistic interactions after concomitant administration of the NMDA ligands with antidepressant drugs were described in the literature (Cieślik et al. 2007; Poleszak et al. 2011, 2014; Szewczyk et al. 2002). A significant reduction in mice immobility in FST has been shown after a joint administration of the sub-therapeutic doses of ifenprodil (an allosteric modulator selectively binding at the NR2B subunit of NMDA receptor (Kew et al. 1996) and imipramine. Increasing of antidepressant-like activity of the NMDA receptor ligands by imipramine has been confirmed in preclinical studies. Use of this TCA together with, e.g., amantadine or memantine (Rogóż 2009; Rogóż et al. 2002, 2004), zinc (Cunha et al. 2008; Szewczyk et al. 2002, 2009), L-701,324 and d-cycloserine (Poleszak et al. 2011) enhanced the duration of animals’ active behavior in the FST and TST (tail suspension test). Desipramine, tested in the present study, which belongs to the TCAs, is the active metabolite of imipramine and more strongly reduces the reuptake of noradrenaline (NA) than serotonin (5-HT) (Pużynski 2005). A combination of desipramine and traxoprodil, both at the sub-active doses, exerted a stronger antidepressant-like effect in the FST than either of these drugs administered alone. Similar results were obtained in our previous study which we carried out using another drug from the TCAs—imipramine (15 mg/kg)—in combination with traxoprodil (10 mg/kg) (Poleszak et al. 2016). This would suggest that the use of either selective serotonin reuptake inhibitors (SSRIs) or selective noradrenaline reuptake inhibitors (SNRIs) could potentiate the activity of traxoprodil.

A significant shortening of immobility time in an inescapable situation in the mice FST after a concomitant administration of low and sub-therapeutic doses of some NMDA antagonists and SSRI were described. Ghasemi et al. (2009) and Poleszak et al. (2014) demonstrated a significant interaction between ifenprodil and paroxetine, whereas Rogóż et al. (Rogóż 2009; Rogóż et al. 2004; 2002) showed potentiation of the antidepressant activity of memantine or amantadine when administered together with fluoxetine or venlafaxine. Literature data indicated that the antidepressant-like activity of the NMDA receptor ligand such as L-701,324 and d-cycloserine were also enhanced by SSRIs (Poleszak et al. 2011). Outcomes of the present study are comparable to those obtained by Preskorn et al. (2008) in which potentiation of traxoprodil activity by paroxetine was observed (Poleszak et al. 2016). Poleszak et al. (2016) have administered combination of traxoprodil with other SSRIs (fluoxetine and escitalopram), all in sub-effective doses, and also observed the statistically significant shortening of the immobility time in the FST in mice. The observed synergism in behavioral tests between the NMDA receptor ligands and SSRIs may result from a direct interaction between the glutamatergic and serotonergic systems. Biochemical studies demonstrated that inhibition of the NMDA receptor increased levels of 5-HT in the CNS neurons (Ciranna 2006), e.g., injection of an uncompetitive antagonists of the NMDA receptor—phencyclidine and MK-801—increased the level of 5-HT in rodent brain (Martin et al. 1997; Yan et al. 1997).

The third tested antidepressant that is considered to target serotonin/noradrenaline systems was milnacipran. Lack of influence on the cholinergic system and histamine release makes this drug devoid of TCAs typical side effects (Tran et al. 2003). Wolak et al. (2013) revealed that activation of NMDA receptors (by NMDA administration) attenuated the antidepressant activity of milnacipran (Wolak et al. 2013) and injection of co-agonist at the NMDA receptor—d-serine (Wolosker 2007)—in the FST in mice antagonized the antidepressant-like activity of imipramine, fluoxetine and reboxetine (Poleszak et al. 2011). The observed in present study enhancement of milnacipran activity when co-administered with traxoprodil seem to confirm the results presented by Wolak et al. (2013) that indicated the strengthening of antidepressant action of milnacipran by concomitant application of CGP 27849 (NMDA receptor antagonist). Furthermore, it was also reported that milnacipran reduced the activity of the NMDA receptors (Kohno et al. 2012).

Besides the serotonergic and noradrenergic neurotransmission, the dopaminergic pathways may be involved in the pathomechanism of depressive disorders (Iversen 2005; Randrup and Braestrup 1977). The dopaminergic system is associated with serotonergic, GABAergic, cholinergic, and glutamatergic neurotransduction. Interactions between these neural transmitters systems contribute to changes in their mutual signaling. Accordingly, in our research norepinephrine–dopamine reuptake inhibitor (NDRI)—bupropion (Cooper et al. 1994; Sennfelt et al. 2011; Wilkes 2006)—was included. Also in this case, a reduction in the duration of immobility in the FST in the group receiving bupropion with traxoprodil was noticed. It should be stressed that the active mice’ behavior observed in FST after joint administration of traxoprodil with all tested antidepressants was not associated with changes in locomotor activity of animals.

Pharmacokinetic studies allow an assessment of the type of interaction (pharmacokinetic or pharmacodynamic) between the various drugs used during polytherapy. Based on the results of these studies, the effect of one drug on the process of absorption, distribution, metabolism, and elimination of the other drug/drugs used simultaneously may be evaluated. As a result of such interactions changes in concentrations of an active substance in the target tissue may be observed. Due to a high probability of the occurrence of interactions between traxoprodil and the tested agents, the concentrations of antidepressants and traxoprodil in murine brain were determined. To our knowledge, this is the first study in which an attempt to assess the interactions between traxoprodil and desipramine, paroxetine, milnacipran or bupropion was made. The likelihood of pharmacokinetic interaction between traxoprodil and tested antidepressants is high as traxoprodil is metabolized by cytochrome P450, as is the case with vast majority of drugs, including antidepressants (Johnson et al. 2003). The metabolism of traxoprodil is mainly mediated by CYP2D6 (Johnson et al. 2003), which also participates in the metabolism of TCAs and SSRIs (Pużynski 2005). Some of them (e.g., paroxetine or fluoxetine) are also strong inhibitors of this enzyme (Sproule et al. 1997).

Our results suggest that the interactions between traxoprodil and all studied antidepressants are pharmacokinetic in nature, as indicated by significant changes in traxoprodil concentrations in the brain tissue when administered with the tested agents. As it has been shown that traxoprodil significantly enhanced levels of bupropion in brains of mice treated concomitantly with bupropion and traxoprodil, the increase in antidepressant-like activity of bupropion observed in the FST was most likely to be, at least in part, also the result of a pharmacokinetic interaction. Bupropion is metabolized to hydroxybupropion by CYP2B6, whereas both bupropion and its primary metabolite are potent inhibitors of the enzyme CYP2D6. This may explain the effect of bupropion on traxoprodil pharmacokinetics; however, up to date no data exist in literature supporting the hypothesis that traxoprodil may inhibit CYP2B6.

In the case of desipramine, paroxetine, and milnacipran, it has been shown that their co-administration with traxoprodil significantly enhances traxoprodil concentrations in brain tissue with no effect of this drug on pharmacokinetics of the studied antidepressant drugs. Taken together, the results obtained in this study may indicate that traxoprodil has an impact on bupropion metabolism or it may facilitate its penetration to brain, leading to an augmentation in bupropion concentrations in its site of action, whereas desipramine, paroxetine, milnacipran, and bupropion may exert the same effect on traxoprodil pharmacokinetics. Obviously, it could not be conceivable that the noted shortening of the immobility time in the FST after combined administration of traxoprodil and the tested drugs is also a result of the pharmacodynamic interaction, like augmentation of the concentration of monoamines or receptor changes in the CNS. Accordingly, it is necessary to extend studies to more biochemical and molecular detailed analysis in order to accurately assess the type of occurring interactions.

## Conclusions

In summary, concomitant administration of traxoprodil with desipramine, paroxetine, milnacipran or bupropion at non-effective doses significantly affects the animals’ behavior in the FST and, the important thing is, these changes are not due to the altered locomotor activity of mice. All these results may indicate that activation of the NMDA receptor is meaningful for the antidepressant-like effect of antidepressant drugs observed in the mice FST. The results of an attempt to assess the nature of the interaction between traxoprodil and examined antidepressants show that there are significant interactions in the pharmacokinetic phase between studied drugs. The influence of the NMDA receptor antagonist—traxoprodil—on the effectiveness of conventionally used antidepressant drugs has highlighted new directions in research and brought to attention other possibilities in therapeutic treatment of affective disorders.
